# Exhaled Volatile Organic Compounds during Inflammation Induced by TNF-α in Ventilated Rats

**DOI:** 10.3390/metabo10060245

**Published:** 2020-06-15

**Authors:** Frederic W. Albrecht, Felix Maurer, Lukas M. Müller-Wirtz, Michaela H. Schwaiblmair, Tobias Hüppe, Beate Wolf, Daniel I. Sessler, Thomas Volk, Sascha Kreuer, Tobias Fink

**Affiliations:** 1 CBR—Center of Breath Research, Department of Anaesthesiology, Intensive Care and Pain Therapy, Saarland University Medical Center and Saarland University Faculty of Medicine, 66421 Homburg/Saar, Germany; felix.maurer@uks.eu (F.M.); lukas.wirtz@uks.eu (L.M.M.-W.); michaela.schwaiblmair@t-online.de (M.H.S.); tobias.hueppe@uks.eu (T.H.); beate.wolf@uks.eu (B.W.); thomas.volk@uks.eu (T.V.); sascha.kreuer@uks.eu (S.K.); tobias.fink@uks.eu (T.F.); 2 Department of Outcomes Research, Anesthesiology Institute, Cleveland Clinic, Cleveland, OH 44195, USA; ds@or.org

**Keywords:** VOC, TNF-α, inflammation, breath analysis, multicapillary-column ion mobility spectrometry, MCC-IMS, anesthesia

## Abstract

Systemic inflammation alters the composition of exhaled breath, possibly helping clinicians diagnose conditions such as sepsis. We therefore evaluated changes in exhaled breath of rats given tumor necrosis factor-alpha (TNF-α). Thirty male Sprague-Dawley rats were randomly assigned to three groups (n = 10 each) with intravenous injections of normal saline (control), 200 µg·kg^−1^ bodyweight TNF-α (TNF-α-200), or 600 µg·kg^−1^ bodyweight TNF-α (TNF-α-600), and were observed for 24 h or until death. Animals were ventilated with highly-purified synthetic air to analyze exhaled air by multicapillary column–ion mobility spectrometry. Volatile organic compounds (VOCs) were identified from a database. We recorded blood pressure and cardiac output, along with cytokine plasma concentrations. Control rats survived the 24 h observation period, whereas mean survival time decreased to 22 h for TNF-α-200 and 23 h for TNF-α-600 rats. Mean arterial pressure decreased in TNF-α groups, whereas IL-6 increased, consistent with mild to moderate inflammation. Hundreds of VOCs were detected in exhalome. P-cymol increased by a factor-of-two 4 h after injection of TNF-α-600 compared to the control and TNF-α-200. We found that 1-butanol and 1-pentanol increased in both TNF-α groups after 20 h compared to the control. As breath analysis distinguishes between two doses of TNF-α and none, we conclude that it might help clinicians identify systemic inflammation.

## 1. Introduction

Inflammation is a complex biological response to infectious and non-infectious stimuli [1]. Detection is difficult, because symptoms are non-specific and highly variable. There is no definitive laboratory test, although there are many non-specific inflammatory markers. Tumor necrosis factor alpha (TNF-α) plays a key role and activates and modulates inflammation. This pro-inflammatory cytokine is released by macrophages, lymphocytes, natural killer cells, and epithelial cells. TNF-α has complex interactions in apoptosis and inflammation [2,3] and activates several important inflammatory pathways [4].

Multicapillary column ion-mobility spectrometry (MCC-IMS) is able to detect volatile organic compounds (VOCs) in a range from ng/L to pg/L which are associated with several pathologies in both animals and humans. For example, exhaled gases are potential diagnostic tools for lung cancer [5,6,7], chronic obstructive pulmonary disease [8], and hemorrhagic shock [9]. Breath analysis even allows detecting gastric cancer, differentiating stages of precancerous gastric lesions [10], and helps distinguish between patients with malignant pleural mesothelioma and healthy subjects [11].

Guamán et al. ventilated rats after injection of lipopolysaccharide (LPS) or saline. The authors distinguished treatment and control animals using IMS and solid phase microextraction-gas chromatography/mass spectrometry (SPME-GC/MS) and concluded that IMS might be a useful point-of-care tool for detection of inflammation [12]. Changes in exhaled volatile organic compounds (VOCs) have also been used to detect polymicrobial sepsis and inflammation in rats [13]. Breath analysis of volatile organic compound (VOCs) thus has potential for detecting and tracking various metabolic and pathologic conditions.

TNF-α is an especially important cytokine in sepsis and other inflammatory conditions and promotes changings in various metabolic pathways. We therefore tested the hypothesis that injection of TNF-α provokes specific changes in the exhalome of rats that might be diagnostic for sepsis and inflammation.

## 2. Results

### 2.1. Survival Time and Hemodynamic Parameters

**Figure 1:**
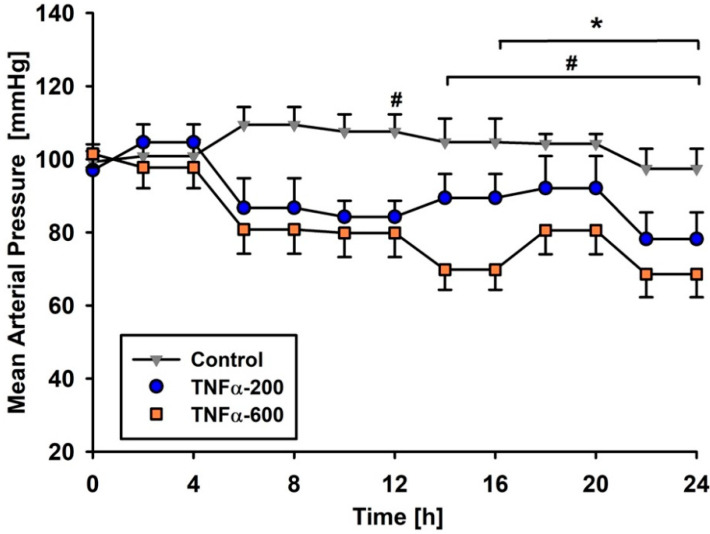
Mean arterial pressure. * *p* < 0.05 control group vs. TNF-α-200; # *p* < 0.05 control group vs. TNF-α-600. Mean arterial pressure was significantly lower in TNF-α-600 after 4 h compared to the corresponding baseline. Results presented as means ± SEMs. N = 10 per group at the beginning of the experiment.

All control rats survived the observation period of 24 h. TNF-α-200 reduced mean survival time to 21.6 ± 2.2 h; CI 95% 17.3–24.0 h, and TNF-α-600 reduced survival time to 22.8 ± 0.6 h; CI 95% 18.0–24.0 h). However, neither reduction was statistically significant (Supplementary Table S1). Hemodynamic responses remained stable in the rats given vehicle, whereas mean arterial blood pressure decreased significantly in both TNF-α groups, with hypotension progressively worsening over time (Figure 1). Cardiac output increased in all groups throughout observation when compared to the adaptation period before administration of trial medication, which was defined as corresponding baseline (Supplementary Table S1).

### 2.2. Blood Gas Analysis

Blood gas partial pressures and pH were initially similar in each group. After 12 h, pH was significantly lower in TNF-α-200 rats compared to control animals and differed significantly at the end of the observation period when compared to baseline. The partial pressure of oxygen remained essentially unchanged throughout in all groups. The partial pressure of carbon dioxide decreased significantly in all groups temporarily, but was again similar to initial values after 24 h. In contrast, blood lactate concentration increased and base excess decreased significantly in both TNF-α groups when compared to control rats and baseline (Supplementary Table S1).

IL-6 plasma concentrations significantly differed between the control group and both TNF-α groups after 12 h. IL-10 did not differ significantly among the groups (Table 1).

### 2.3. Multicapillary Column Ion-Mobility Spectrometry in Rats

**Figure 2:**
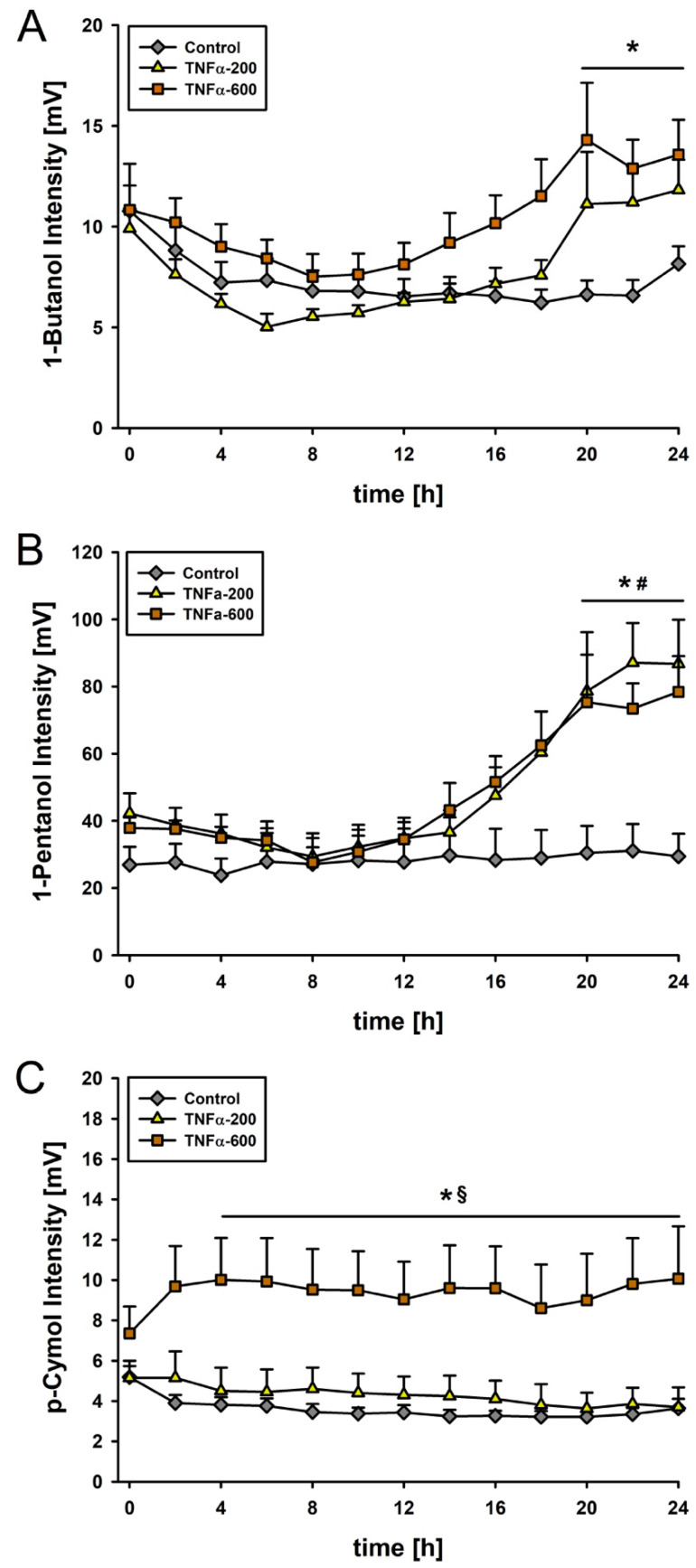
(**a**) 1-butanol during observation period. * *p* < 0.05 for TNF-α-200 and TNF-α-600 vs. control group; (**b**) 1-pentanol: *# significant increase of 1-pentanol intensity for both TNF-α groups vs. baseline and vs. control group; (**c**) Course of p-cymol intensity: * *p* < 0.05 TNF-α-600 vs. control group. § *p* < 0.05 TNF-α-600 vs. TNF-α-200. Data presented as means ± SEMs. N = 10 per group at the beginning of the experiment.

About a hundred VOCs were detected by MCC-IMS. At the beginning of the observation period, each group generated comparable peaks. We found that 1-butanol increased in TNF-α-200 by about 40% and in TNF-α-600 by about 60% compared with the control group after 20 h. In both TNF-α groups, intensity of 1-pentanol was twice as high as in the control group after 20 h. A dose-dependent effect of TNF-α was detected for p-cymol. Specifically, 4 h after injection of 600 µg/kg/BW TNF-α, p-cymol doubled compared to the control and TNF-α-200 (Figure 2).

### 2.4. Multicapillary Column Ion-Mobility Spectrometry of TNF-α after Vaporization

The vaporization of TNF-α with a test gas generator did not facilitate detection of 1-butanol, p-cymol, or 1-pentanol.

## 3. Discussion

We used intravenous TNF-α-injections to induce a mild to moderate systemic inflammatory response in rats. We were able to identify exhaled markers for inflammation, differing not only to healthy controls but also over time. These findings may help to facilitate an early and non-invasive diagnosis of sepsis in the future.

Our experimental model of systemic inflammation was based on previous work by Tracey et al. [14]. Our rats developed consistently hypotension and metabolic acidosis after intravenous doses of 200 or 600 µg·kg^−1^ BW TNF-α, confirming systemic inflammatory response. Cardiac output significantly increased in all groups with no differences to healthy controls. Our results are thus consistent with the findings by Tracey et al. [14], and can be interpreted as mild to moderate systemic inflammation caused by TNF-α.

Patel et al. demonstrated in 1995 that IL-6 concentration is highly predictive for serious complications and mortality in patients with severe intra-abdominal sepsis. IL-6 was significantly higher, by a factor-of-five, in non-survivors [15]. In our study, IL-6 concentrations were significantly greater, roughly by a factor-of-two, in both TNF-α groups than in control rats after 12 h, suggesting that TNF-α provoked a moderate inflammation. Survival time was reduced in both our TNF-α groups, although not significantly so.

The concentration of the alkanol 1-butanol increased about 40% after administration of TNF-α-200, and about 60% after TNF-α-600. Wu and colleagues analyzed, in 2017, the effect of 1-butanol on human neutrophils after lipopolysaccharide (LPS) induced inflammation. They concluded that n-butanol moderates LPS-induced down-regulation of signaling pathways and stimulates myeloperoxidase activity in neutrophils [16]. Additionally, Dong et al. reported, in 2015, a significant reduction of the inflammatory cytokines TNF-α and IL-6 after pre-treatment with 1-butanol in a rodent model of LPS-induced inflammation. [17]. Cai et al. showed, in 2014, a dose-dependent reduction of the pro-inflammatory mediators nitric oxide, prostaglandin E2, and the cytokines TNF-α, IL-1β, and IL-6 in LPS-stimulated macrophages in vitro [18]. In our study, 1-butanol increased in both TNF-α groups compared to the control group, presumably consequent to a generalized inflammatory response. The anti-inflammatory effects of 1-butanol have therefore been demonstrated in several studies; nonetheless, understanding of the signaling pathways and their mechanisms remain poor.

Yamada et al. compared, in 2017, the exhalome from patients with idiopathic pulmonary fibrosis (IPF) with that from healthy subjects. p-cymol was significantly lower in patients with IPF compared to the control. Overall, p-cymol was related to vital capacity, predicted percentage of vital capacity, and forced vital capacity [19]. In a rodent model of LPS-induced lung injury, Xie et al. evaluated, in 2012, the effect of intraperitoneal pre-treatment with p-cymol [20]. Preconditioning with p-cymol caused a significant reduction of the pro-inflammatory cytokines TNF-α, IL-1β, and IL-6. They also observed reduced infiltration of inflammatory cells and reduced myeloperoxidase activity in the lung tissue, suggesting that p-cymol protects lungs from LPS-induced injury [20]. Available data thus suggest that p-cymol moderates some harmful effects of inflammatory cytokines. In the high-dose TNF-α group, p-cymol increased significantly compared to the control group and the low-dose TNF-α group. These results support the suggestion that p-cymol influences inflammation. Signaling pathways and origin of p-cymol in the exhalome remain unclear and need further investigation.

Marecaux et al. recommended, in 1996, using repetitive lactate concentrations to predict survival of septic patients. Lactate concentrations were more useful than TNF-α and IL-6, because cytokine concentrations are so variable [21]. In our high-dose TNF-α rats, exhaled p-cymol increased even earlier than lactate concentration, suggesting that p-cymol might facilitate early detection of inflammation and sepsis. We found that 1-pentanol also increased in response to TNF-α, suggesting that it too might be an early marker of inflammation.

The vaporization of the TNF-α solution with a HovaCAL test gas generator did not identify 1-butanol, p-cymol, or 1-pentanol as volatile ex vivo products of TNF-α. Consequently, the changes in the exhalome are caused by inflammatory or metabolic changes, rather than by components of the trial medication.

We used a moderate model of inflammation to analyze early changes in the exhalome. Alterations during fulminant and critical stages have yet to be well characterized. The origin of analyzed VOCs, as well as their signaling pathways, remain unclear.

We conclude that 1-butanol, p-cymol, and 1-pentanol are potential VOCs for diagnosis of inflammatory processes or sepsis. Further investigations are needed to determine the origin of these compounds, and the extent to which they are sensitive and specific markers.

## 4. Materials and Methods

### 4.1. Animals

With approval of the Animal Care and Use Committee (LGV), experiments were conducted according to the German Animal Welfare Act (Landesamt für Soziales, Gesundheit und Verbraucherschutz; Saarbrücken; Germany, approval no. 36/2014). Thirty male Sprague-Dawley rats (n = 10 per group) with a body weight between 200 and 300 g (Charles River, Sulzfeld, Germany) were maintained at a temperature of 20 ± 2 °C and a relative humidity of 50 ± 5% in the institutional animal facility. The rats were fasted 12 h before the experiment, with free access to water.

### 4.2. Surgical Procedures of Experimental Animals

Anesthesia was induced with inhaled sevoflurane and maintained by intraperitoneal injection of 60 mg kg^−1^ body weight (BW) pentobarbital. Atracurium was given as necessary to maintain muscle relaxation. Animals were positioned on a heating plate and a stainless steel tracheal canula was inserted to facilitate breathing. The right jugular vein was catheterized, and a continuous infusion of 5 mL kg^−1^ BW h^−1^ of isotonic solution (Sterofundin BG-5, B. Braun, Melsungen, Germany) was given throughout. Heart rate and mean arterial pressure were measured via an arterial catheter in the left femoral artery as described previously [22]. A t-type implantable thermocouple MLT 1405 was inserted into the left carotid artery and connected with a Cardiac Output Pod ML313 (AD Instruments Ltd., Oxford, UK) to monitor body temperature and cardiac output. Physiological parameters were measured and analyzed with LabChart 7 using a PowerLab 8/35 (AD Instruments Ltd., Oxford, UK).

Animals were mechanically ventilated with highly purified synthetic air (Air Liquid, Ludwigshafen, Germany) by a small animal ventilator (KTR-5, Hugo Sachs Elektronik, March-Hugstetten, Germany) as previously described [23]. Ventilation was maintained for 24 h or until death. The ventilator was initially set to a tidal volume of 8 mL kg^−1^ BW, respiratory rate 65 breaths/min, inspiration ratio 45%, and positive end expiratory pressure of 2 cm H_2_O, and adapted as necessary to maintain partial pressure of oxygen between 75 and 150 mmHg and carbon dioxide between 32 and 45 mmHg, as measured by blood gas analysis (Radiometer ABL 800 Basic, Willich, Germany).

### 4.3. Experimental Protocol

Under randomized and blinded conditions, rats were assigned into a control group (saline as solvent), a low concentration TNF-α group (200 µg·kg^−1^ BW), and a high concentration TNF-α group (600 µg·kg^−1^ BW). Animals were randomized in groups using a random number generator (Excel 2010; Microsoft, Redmond, WA, USA). After an adaption period lasting 30 min, which was defined as baseline, trial medications were injected, and the observation period began. Arterial blood (0.2 mL) was sampled every 4 h for blood gas analysis. Cardiac output was assessed at each blood sampling interval using thermodilution technique by injection of 0.2 mL cold saline into the venous catheter. The thermodilution curve was collected by LabChart 7 and cardiac output was calculated. Blood samples (0.5 mL) for enzyme-linked-immunosorbent assay (ELISA) of IL-6 and IL-10 were taken before administration of trial medications and 12 h afterwards.

### 4.4. MCC-IMS Measurement

A Breath Discovery MCC-IMS (B&S Analytik, Dortmund, Germany) with a 550 MBq ^63^Ni ionization source, with an electrical field strength of 300 Vcm^−1^ and an OV-5 MCC (Multichrom, Novosibirsk, Russia), was utilized to measure volatile organic compounds. A sample of 10 mL of exhaled air was aspirated every 20 min from the expiratory line. Additional technical parameters and experimental setup have been previously described [9,13,22,23].

A comparative analysis was executed with a test gas generator (HovaCAL 4836-VOC, IAS GmbH, Oberursel, Germany). TNF-α was vaporized at 100 °C and a relative humidity of 100% and measured with the MCC-IMS.

The chromatograms and the peak intensities of the vaporized trial medication and the exhalome were compared and analyzed using VisualNow (Version 3.6, B&S Analytik, Dortmund, Germany). Signals above a threshold of 1 mV (five times background noise) were defined as peaks. Peaks were characterized according to retention time (RT), drift time (1/K_0_ value), and intensity. Peak identification was established with MIMA software (version 1.1.2) by an automatic comparison of the peaks with the BS-MCC/IMS-analytes database (version 1209, B&S Analytik, Dortmund, Germany). The database includes the RT, the inverse reduced ion mobility, and the radii representing the typical peak expansion for several analytes.

### 4.5. Cytokine Assay

IL-6 and IL-10 plasma concentrations were measured with enzyme-linked immunosorbent assay (ELISA) before administration of trial medications and 12 h after administration (ELISA Antibodies BD OptEIA; BD Biosciences Pharmingen, SD, CA). An ELISA microplate Reader EL 800 was used for the measurement of the extinctions at 450/620 nm and analysis was performed with Gen5TM (version 1.0, BioTek, Bad Friedrichshall, Germany). Calibration standards and positive controls were routinely measured with each 96-wells microplate.

### 4.6. Data Processing and Statistical Analysis

Statistical analysis was conducted with SigmaPlot (version 12.5; Systat Software, Erkrath, Germany). Based on our previous experience, and an ANOVA sample size calculation, we estimated that 10 rats per group would provide an alpha of 0.05 and a power of 0.8. Survival time was analyzed using a log-rank model according to Kaplan–Meier. Data were tested for distribution of normality (Kolmogorov-Smirnov test). Intergroup comparisons were performed using one-way-ANOVA for normally distributed data, or in case of not normally distributed data, one-way ANOVA on ranks was used, followed by Holm-Sidak’s or Dunn´s post-hoc testing. Longitudinal comparisons were similarly performed but with repeated-measures ANOVA. *p* < 0.05 was considered statistically significant.

## Figures and Tables

**Table 1:** Plasma cytokine concentrations at 12 h.

Groups	IL-6 [pg/mL]		IL-10 [pg/mL]	
	0 h	12 h	0 h	12 h
Control group	289 ± 96	294 ± 94	21 ± 66	20 ± 69
TNF-α-200	312 ± 41	577 ± 1170 *	19 ± 40	33 ± 71
TNF-α-600	275 ± 38	610 ± 1262 *	2 ± 5	28 ± 94

* *p* < 0.05 vs. control group.

## Supplementary Materials

The following are available online at https://www.mdpi.com/2218-1989/10/6/245/s1, Table S1: Survival rate, blood gas analysis, and cardiac output.
