# Supplementary material for: Exhaled Volatile Organic Compounds during Inflammation Induced by TNF-α in Ventilated Rats
Source: Metabolites. 2020 Jun 15;10(6):245. doi: 10.3390/metabo10060245 (PMC7345252; doi:10.3390/metabo10060245)
Supplement: Supplementary file 1 [file metabolites-10-00245-s001.pdf]

		Baseline	4 h	8 h	12 h	16 h	20 h	24 h
**Survival rate**								
	Control group	10	10	10	10	10	10	10
	TNFα-200	10	10	10	9	8	8	8
	TNFα-600	10	10	10	10	10	9	8
**pH**								
	Control group	7.43 ± 0.05	7.46 ± 0.03	7.48 [#] ± 0.03	7.46 ± 0.04	7.47 ± 0.02	7.46 ± 0.04	7.40 ± 0.05
	TNFα-200	7.43 ± 0.03	7.43 ± 0.08	7.44 ± 0.06	7.36 [*] ± 0.10	7.39 ± 0.06	7.39 ± 0.06	7.26 [*#] ± 0.10
	TNFα-600	7.43 ± 0.04	7.48 ± 0.04	7.44 ± 0.05	7.41 ± 0.06	7.41 ± 0.09	7.44 ± 0.09	7.37 ± 0.09
**Base excess**								
	Control group	1.7 ± 1.8	-0.6 [#] ± 2.1	-0.2 [#] ± 1.2	1.6 ± 1.8	-1.2 [#] ± 1.8	-2.0 [#] ± 2.3	-2.3 [#] ± 2.0
	TNFα-200	1.0 ± 2.4	-1.7 ± 2.7	-7.4 [*#] ± 3.7	-6.8 [*#] ± 6.4	-5.8 [#] ± 4.1	-7.2 [*#] ± 4.1	-8.0 [*#] ± 4.9
	TNFα-600	1.0 ± 1.9	0.1 ± 1.8	-5.6 [*#] ± 3.6	-5.6 [*#] ± 3.2	-8.3 [*#] ± 3.7	-7.6 [*#] ± 4.0	-6.2 [#] ± 4.2
**Lactate [mmol/l]**								
	Control group	0.9 ± 0.3	0.6 ± 0.1	0.9 ± 0.3	0.9 ± 0.2	0.8 ± 0.2	0.7 ± 0.2	1.3 ± 0.9
	TNFα-200	0.9 ± 0.2	1.5 [*#] ± 0.4	1.3 [#] ± 0.5	1.5 [#] ± 0.5	1.3 [*#] ± 0.3	1.3 [#] ± 0.3	1.6 [#] ± 0.3
	TNFα-600	0.7 ± 0.1	1.3 [*#] ± 0.2	1.6 [*#] ± 0.5	2.0 [*#] ± 0.5	1.9 [*#] ± 0.4	1.9 [*#] ± 0.6	2.3 [#] ± 1.0
**Partial pressure of oxygen [mmHg]**								
	Control group	83.6 ± 12.8	85.7 ± 11.5	97.9 ± 16.4	84.9 ± 10.8	94.1 ± 15.3	87.5 ± 15.5	84.8 ± 17.0
	TNFα-200	96.8 ± 2.4	96.4 ± 2.7	111 ± 3.7	103 ± 6.4	108 ± 4.1	107 ± 4.1	78.4 ± 4.9
	TNFα-600	84.3 ± 1.9	96.9 ± 1.8	110 ± 3.6	106 ± 3.2	115 [#] ± 3.7	97.0 ± 4.0	90.0 ± 4.2
**Partial pressure of carbon dioxide [mmHg]**								
	Control group	40.4 ± 7.3	32.7 ± 2.8	31.6 [#] ± 3.3	35.3 ± 4.4	31.1 [#] ± 3.1	30.7 ± 3.7	37.6 ± 4.0
	TNFα-200	36.0 ± 4.6	35.3 ± 9.4	25.2 [#] ± 3.8	31.8 ± 5.0	29.7 ± 4.2	27.5 ± 4.5	39.8 ± 11.5
	TNFα-600	36.2 ± 5.8	32.2 ± 3.9	27.0 [#] ± 4.8	28.5 [*#] ± 3.7	28.5 [#] ± 5.0	27.5 [#] ± 3.2	31.8 ± 7.3

Cardiac Output
[ml/min]

Control group	**92.3** ± *16.3*	**102** ± *31.1*	**105** ± *40.4*	**97.7** ± *14.9*	**106** ± *23.6*	**136** # ± *52.9*	**167** # ± *66.5*
TNFα-200	**82.6** ± *16.2*	**71.8** ± *13.1*	**82.8** ± *15.4*	**98.5** ± *10.1*	**131** # ± *31.4*	**134** # ± *15.5*	**140** # ± *17.1*
TNFα-600	**82.8** ± *9.6*	**85.2** ± *20.1*	**76.9** ± *20.2*	**104** ± *29.3*	**108** ± *25.7*	**111** ± *29.8*	**139** # ± *22.3*

Supplementary Table Survival rate, blood gas analysis and cardiac output: * $p<0.05$ vs. Control group, # $p<0.05$ vs. corresponding baseline.

TNFα-200 = 200µg/kg/BW TNFα, TNFα-600 = 600 µg/kg/BW TNFα. Data are given as means ± CIs
